# Supplementary figures and images for: Peculiar transcriptional reprogramming with functional impairment of dendritic cells upon exposure to transformed HTLV-1-infected cells
Source: PLoS Pathog. 2024 Sep 16;20(9):e1012555. doi: 10.1371/journal.ppat.1012555 (PMC11426526; doi:10.1371/journal.ppat.1012555)

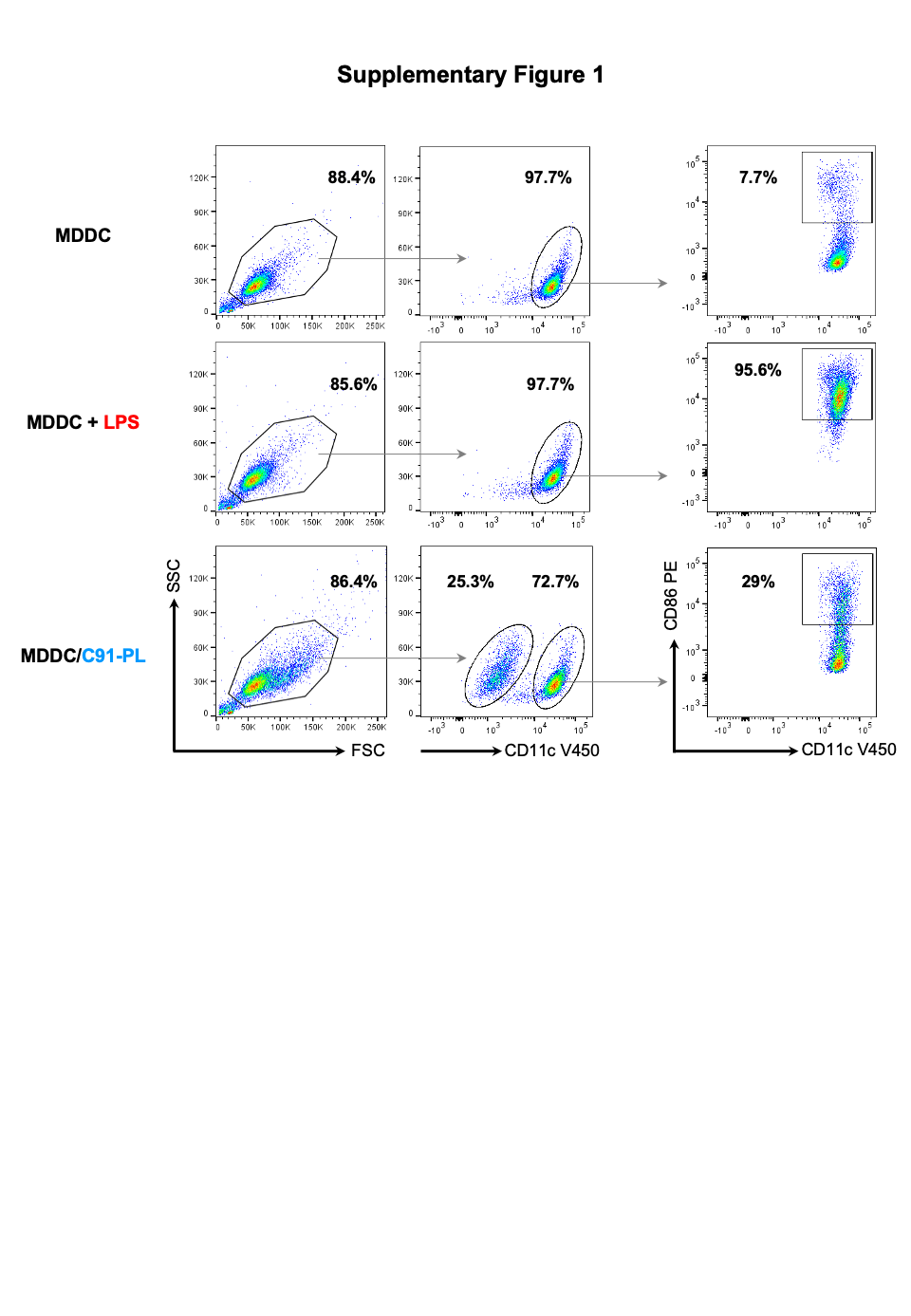

Supplement: S1 Fig — Example of the hierarchical gating strategy used to analyze MDDC phenotype. Control MDDCs (top panel), MDDCs treated with LPS for 24h (middle panel) or MDDCs cocultured with HTLV-1-infected C91-PL T cells (bottom panel) were identified based on their expression of CD11c, which is absent on T cells, and their maturation status was determined by their expression of CD86 (or other maturation or inhibition markers, see S2A–S2F Fig). (TIFF) [file ppat.1012555.s001.tiff]

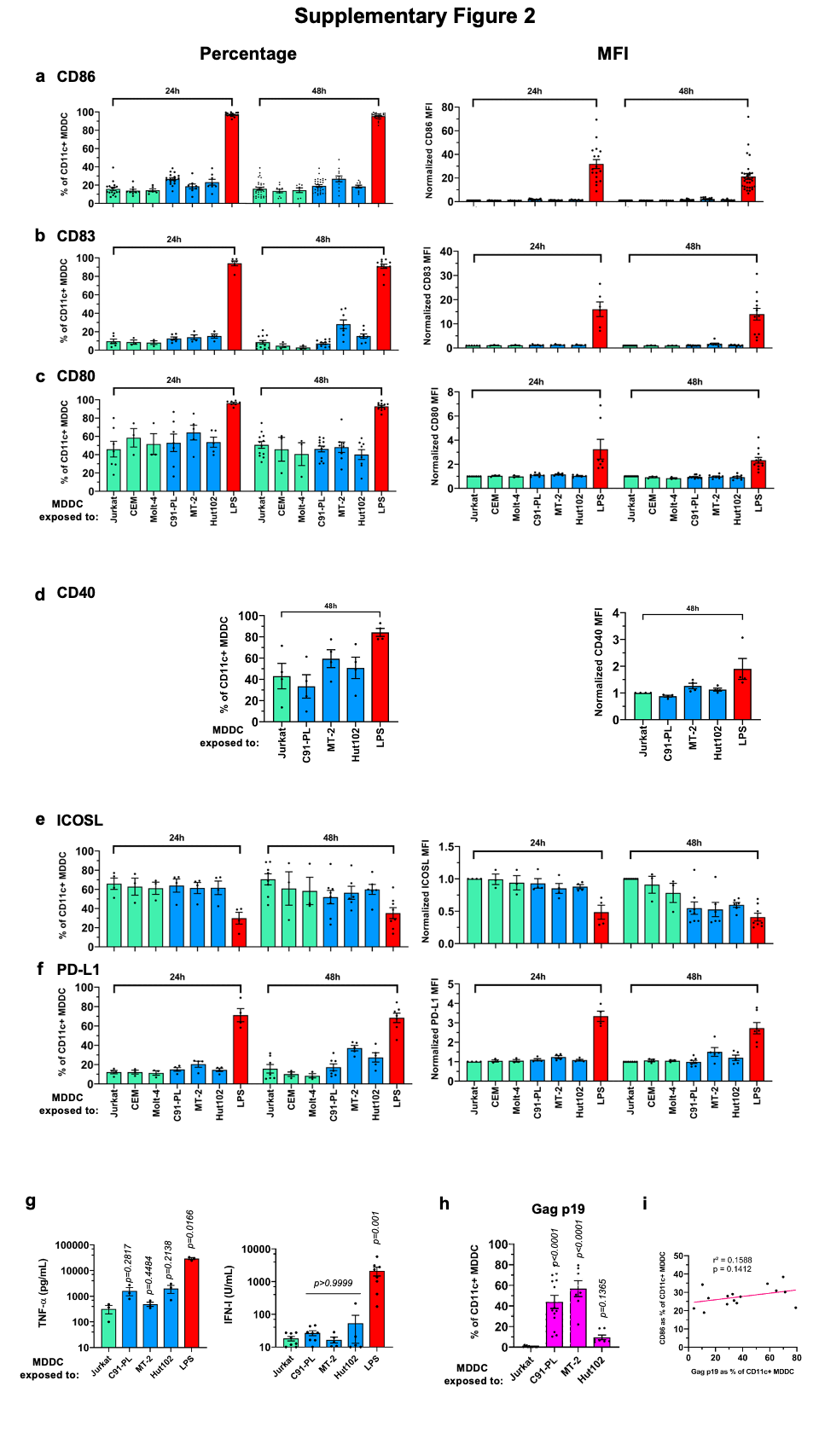

Supplement: S2 Fig — This figure is relative to Fig 1A-1F. MDDCs were cocultured with control uninfected (Jurkat, CEM, Molt-4, green bars), or HTLV-1-infected T cell lines (C91-PL, MT-2, Hut102, blue bars) for 24h or 48h, as indicated. As a control, MDDCs were stimulated with LPS for 24h (“24h” condition), or left untreated for 24h followed by 24h of LPS treatment (“48h” condition, red bars). Flow cytometry analysis after CD11c and CD86 (a), CD83 (b), CD80 (c), CD40 (d), ICOSL (e) and PD-L1 (f) staining. Data are represented as percentage of positive MDDCs among total CD11c+ MDDCs (left), or as normalized MFI (right) for n = 3–31 independent experiments. Detailed statistical analysis is presented in S8 Table. Briefly, data were analysed with ordinary one-way ANOVA or Kruskal-Wallis test, in accordance with the distribution of the dataset. A complementary analysis with cell lines grouped according to infection status (MDDC exposed to non-infected cells: Jurkat, CEM, Molt-4; MDDC exposed to HTLV-1-infected cells: C91-PL, MT-2, Hut102; MDDC stimulated with LPS) was performed with nested one-way ANOVA, as presented in S8 Table. g. Supernatant from the indicated cocultures or from LPS-stimulated MDDCs was collected, and TNF-α (left) and IFN-I (right) were quantified by Luminex and reporter cell assay, respectively. Results from n = 3 or 8–5 independent experiments, respectively. Data were analysed with RM one-way ANOVA or Kruskal-Wallis test, respectively, and with nested one-way ANOVA, grouping cell lines according to the infection status, as presented in S8 Table. h. Viral capture in MDDCs cocultured with Jurkat, C91-PL, MT-2 or Hut102 cells was assessed by Gag p19 staining on CD11c+ MDDCs. The percentage of Gag p19+ MDDCs was determined in n = 7–14 independent experiments. Data were analysed with Kruskal-Wallis test, as presented in S8 Table. i. The percentage of CD86+ MDDCs from repeated experiments was plotted against the percentage of Gag p19+ MDDCs (n = 15), and a linear correlation f [file ppat.1012555.s002.tiff]

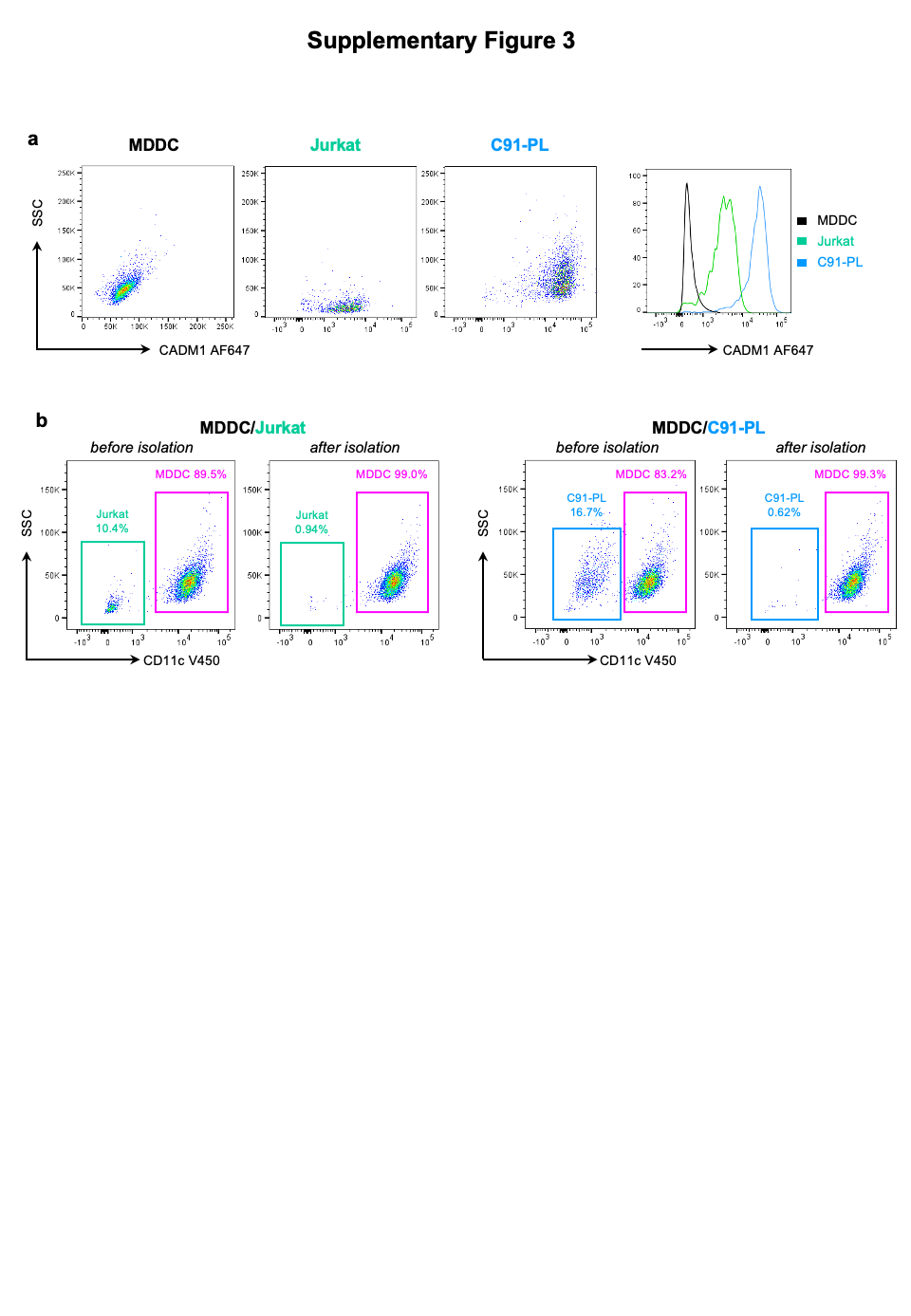

Supplement: S3 Fig — a. CADM1 expression on MDDCs, Jurkat or C91-PL cells. Expression levels in individual cell types were superimposed (right) to show the exclusive expression of CADM1 on T cells compared to MDDCs, with higher expression levels on C91-PL cells compared to Jurkat cells. b. MDDC were cocultured for 24h with Jurkat (left) or C91-PL T cells (right), and cells from each coculture condition were stained for CD11c before or after negative magnetic selection based on CADM1. Representative flow cytometry plots are shown. The percentages of T cells (Jurkat, green, or C91-PL, cyan) and of MDDCs (magenta), before or after isolation of MDDCs, are indicated. (TIFF) [file ppat.1012555.s003.tiff]

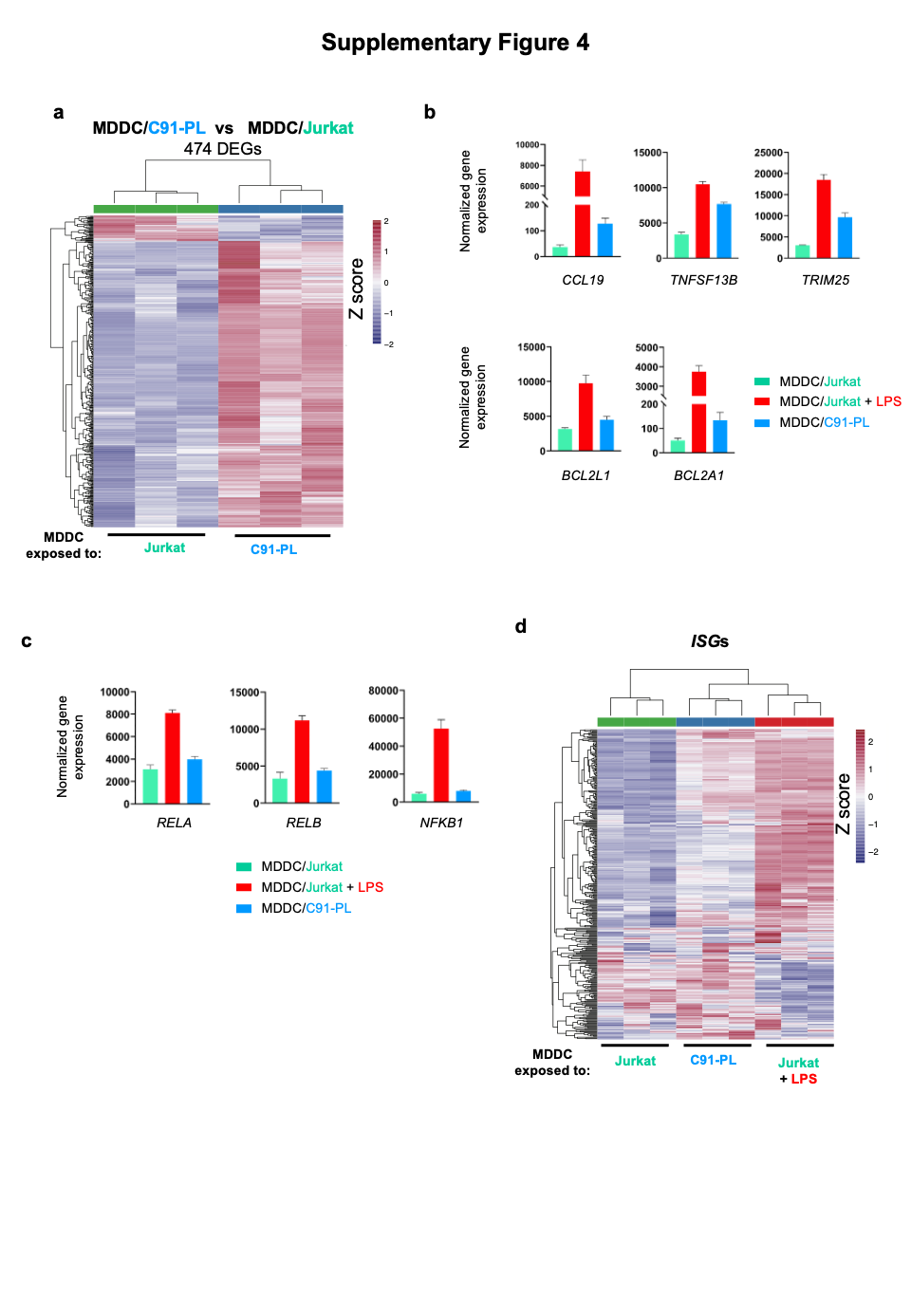

Supplement: S4 Fig — This figure is relative to Fig 2. a. Heatmap of the 474 total DEGs in C91-PL-exposed MDDCs compared to Jurkat-exposed MDDCs. b. DESeq2 normalized counts for CCL19, TNFSF13B, TRIM25, BCL2L1 and BCL2A1 across samples. c. DESeq2 normalized counts for RELA, RELB and NFKB1 across samples. d. Heatmap of ISGs expression across samples. (TIFF) [file ppat.1012555.s004.tiff]

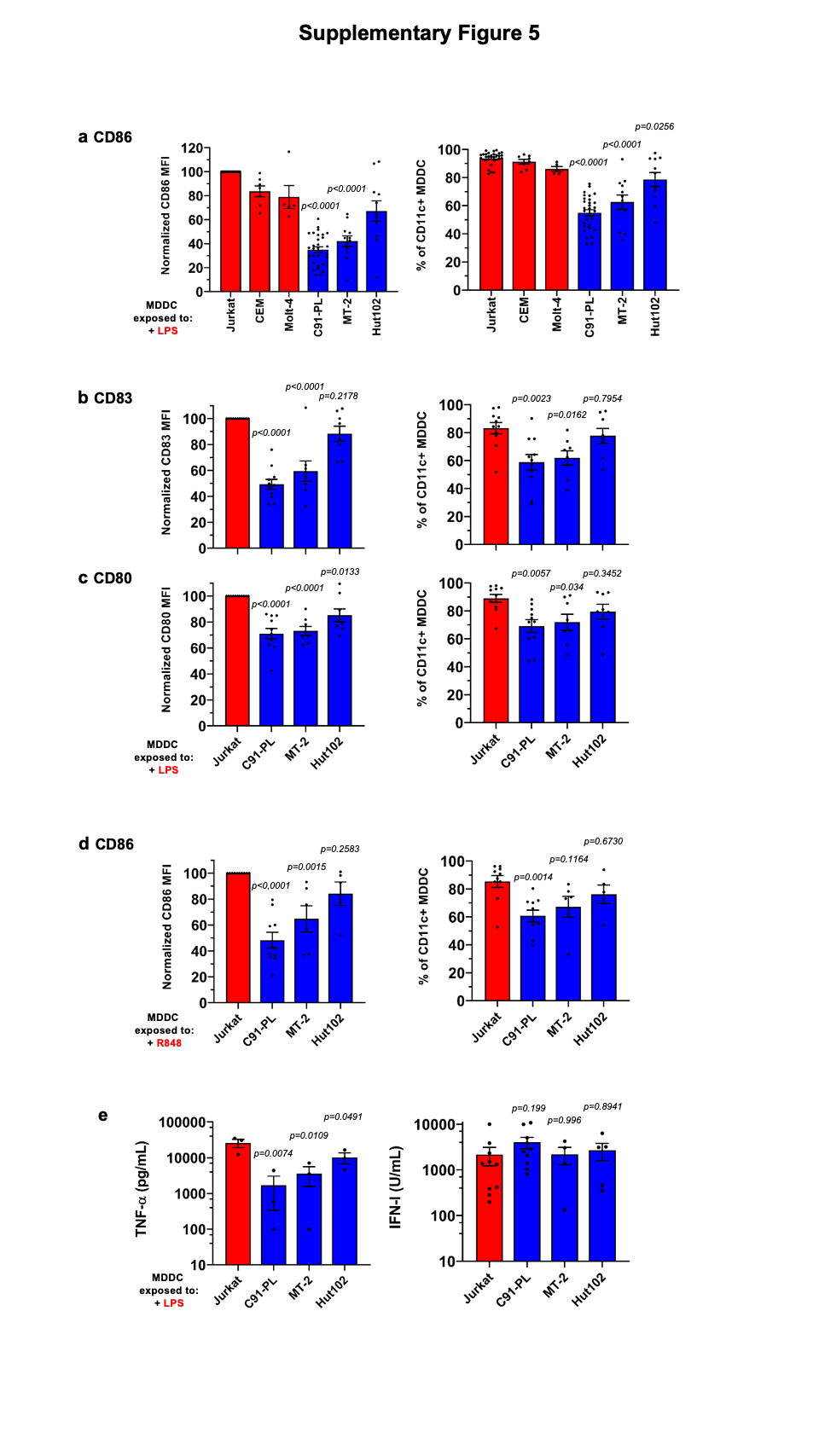

Supplement: S5 Fig — This figure is relative to Fig 3. MDDCs were cocultured with uninfected (Jurkat, CEM, Molt-4, red bars) or HTLV-1-infected T cells (C91-PL, MT-2, Hut102, dark blue bars) for 24h, before restimulation with LPS (a-c, e) or R848 (d) for an additional 24h. a-d. Flow cytometry analysis after CD11c and CD86, CD83 and CD80 staining. The normalized MFI (left) and percentage of positive MDDCs (right) was determined in n = 5–30 independent experiments. Detailed statistical analysis is presented in S8 Table. Briefly, data were analyzed with ordinary one-way ANOVA or Kruskal-Wallis test, in accordance with the distribution of the dataset. e. Supernatant from the indicated cocultures was collected after LPS restimulation, and TNF-α (left) and IFN-I (right) concentrations were quantified for n = 10 or 4–5 independent experiments, respectively. Data were analyzed using RM one-way ANOVA or ordinary one-way ANOVA, respectively, as presented in S8 Table. (TIFF) [file ppat.1012555.s005.tiff]

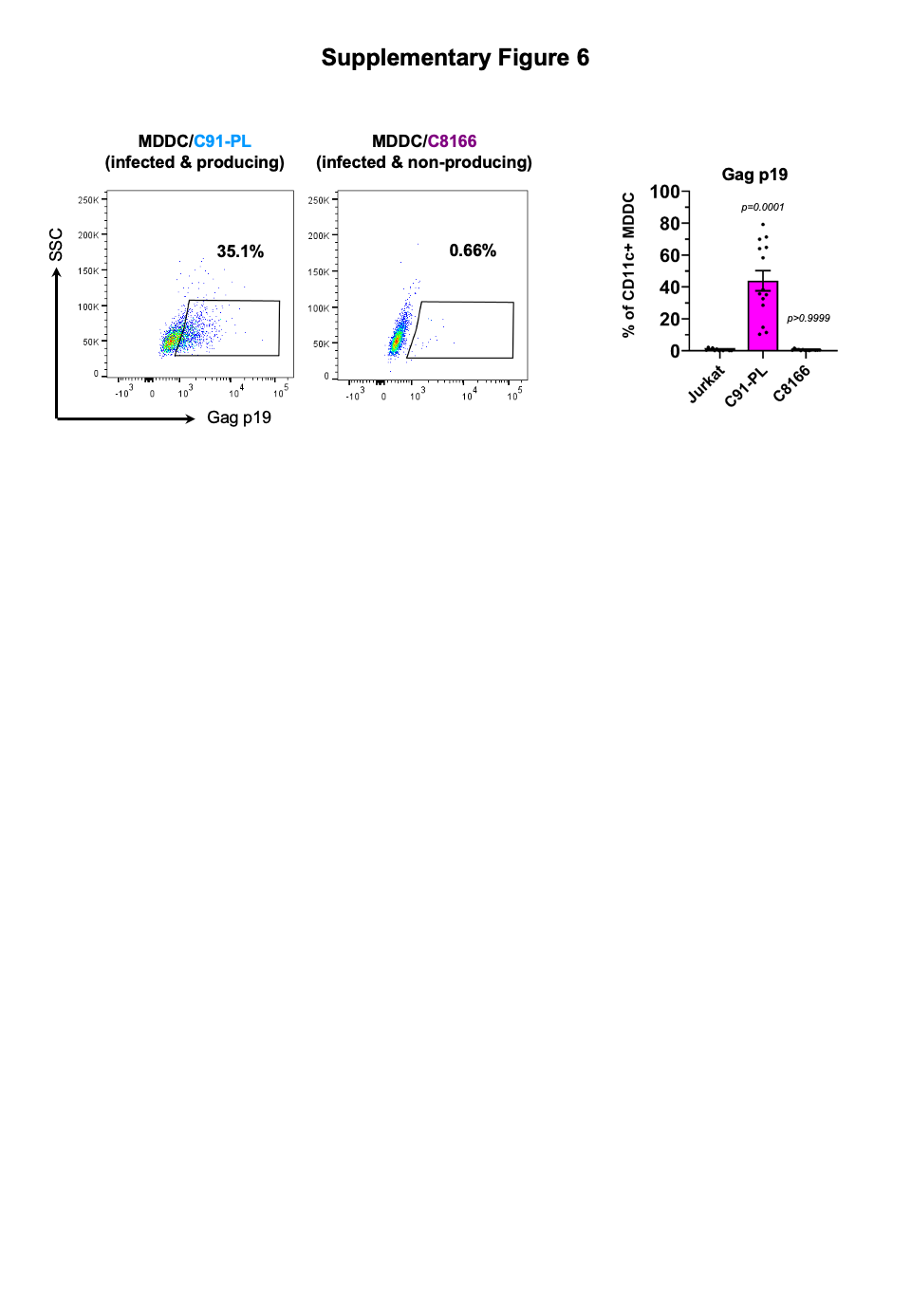

Supplement: S6 Fig — Viral capture in MDDCs cocultured with C91-PL or C8166 T cells was assessed after 24h of coculture by Gag p19 staining on CD11c+ MDDCs. The percentage of Gag p19+ MDDCs among total CD11c+ MDDCs was determined. Left: representative experiment. Right: Results from n = 14 independent experiments. Data were analysed with Friedman test, as presented in S8 Table. (TIFF) [file ppat.1012555.s006.tiff]

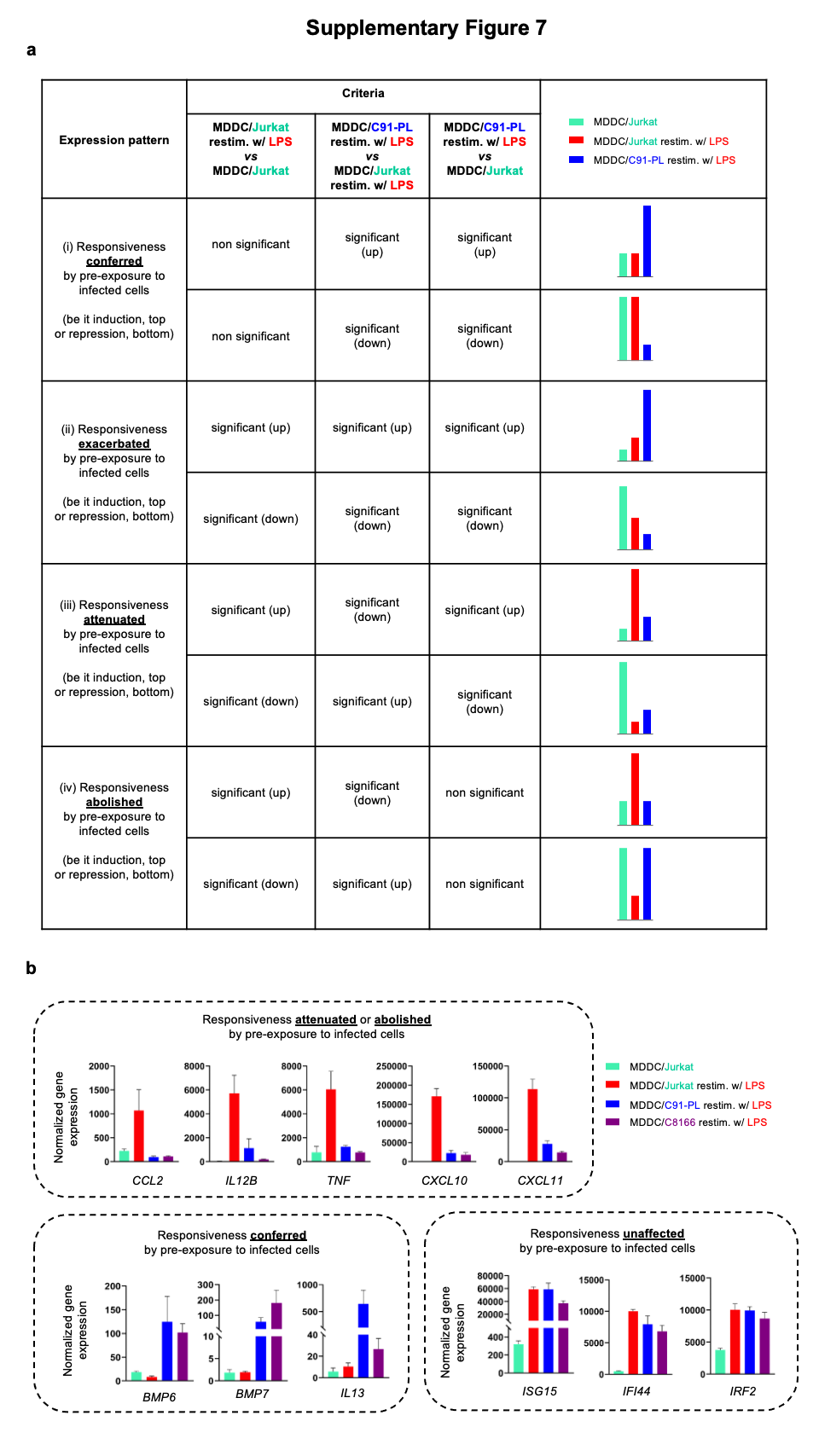

Supplement: S7 Fig — This figure is relative to Fig 4. a. Recapitulative table summarizing the different gene responsiveness patterns observed in the dataset (see Material and Methods for details). Genes were classified by comparing the DEG lists retrieved from the 3 comparisons indicated on the top of the table (criteria). A theoretical expression pattern across samples is illustrated in the right column. b. DESeq2 normalized counts across samples of selected genes showing an “attenuated or abolished” (top), “conferred” (middle) or “unaffected” (bottom) responsiveness pattern. (TIFF) [file ppat.1012555.s007.tiff]

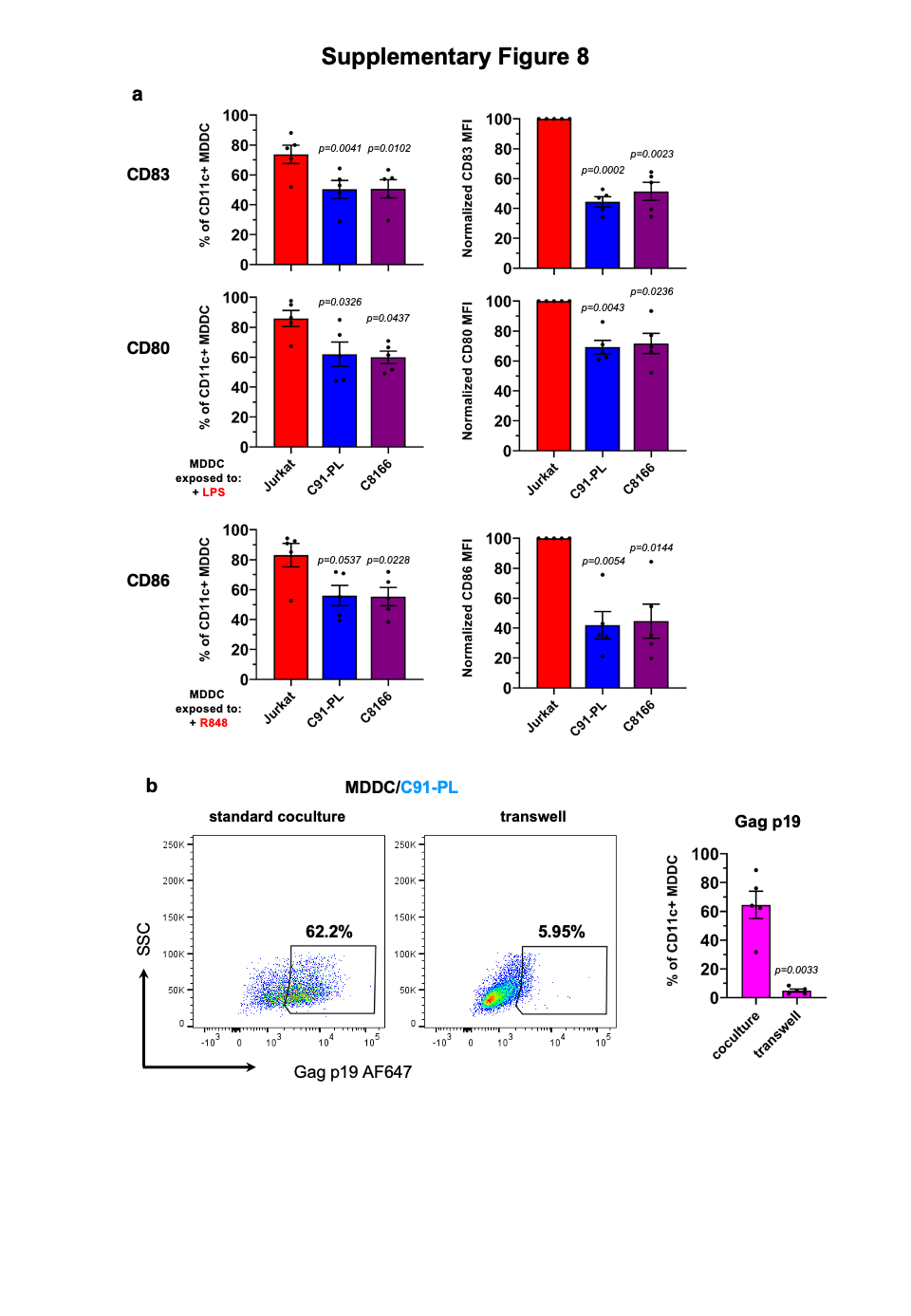

Supplement: S8 Fig — This figure is relative to Fig 5. a. Flow cytometry analysis after staining for CD11c and CD83 (top), CD11c and CD80 (middle), or CD11c and C86 (bottom). Data are represented as percentage of CD83+ (CD80+ or CD86+ respectively) MDDCs (left), or as normalized MFI of CD83 (CD80 or CD86 respectively) (right), with the MFI in re-stimulated Jurkat-pre-exposed MDDCs set to 100, and summarize n = 5 independent experiments. Red: Jurkat-pre-exposed MDDC restimulated with LPS or R848; dark blue: C91-PL pre-exposed MDDC restimulated with LPS or R848; purple: C8166 pre-exposed MDDC restimulated with LPS or R848. Detailed statistical analysis is presented in S8 Table. Briefly, data were analysed with RM one-way ANOVA Friedman test, in accordance with the distribution of the dataset. b. Representative cytometry plots (left) showing the viral capture in MDDCs cocultured with C91-PL T cells, in the absence (standard coculture) or presence (transwell) of a transwell insert (0.4μm pore diameter), as assessed by Gag p19 staining on CD11c+ MDDCs. The quantification of data from n = 5 independent experiments are summarized (right). Data were analysed with a paired t-test, as presented in S8 Table. (TIFF) [file ppat.1012555.s008.tiff]

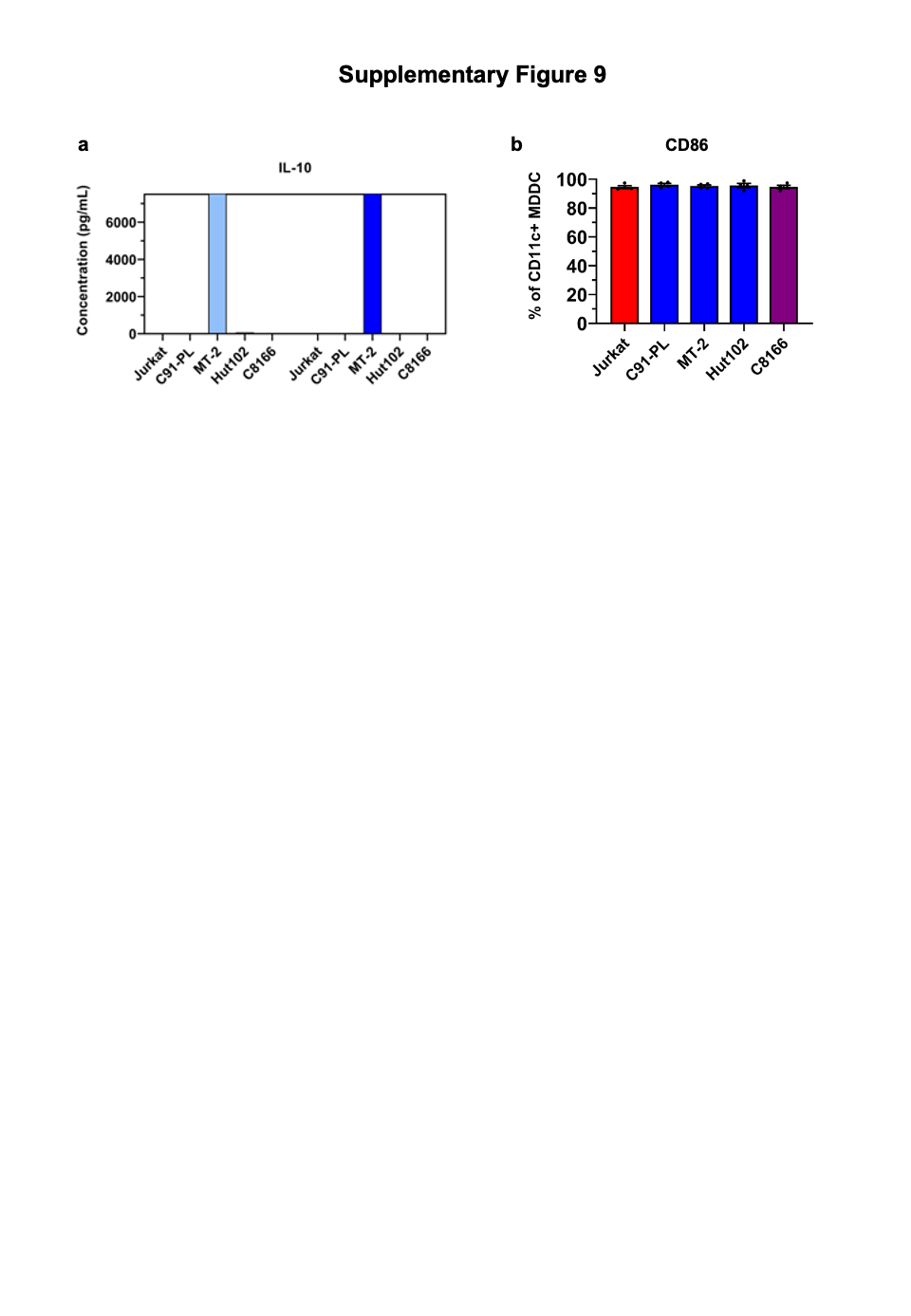

Supplement: S9 Fig — a. Quantification by Luminex of IL-10 levels in supernatants of MDDCs cocultured with uninfected T cells (Jurkat), or with HTLV-1-infected T cells (C91-PL, MT-2, Hut102, C8166), followed or not by LPS stimulation (n = 1 experiment). b. Uninfected (Jurkat, red) or HTLV-1-infected T cells (C91-PL, MT-2, Hut102, dark blue; or C8166, purple) were cultured for 24h. Conditioned medium was collected and added on naive MDDCs for 24h, before restimulation with LPS for an additional 24h. Quantification results from n = 4 independent experiments of flow cytometry data for CD11c and CD86 staining are presented. Data were analysed with RM one-way ANOVA, as presented in S8 Table. (TIFF) [file ppat.1012555.s009.tiff]

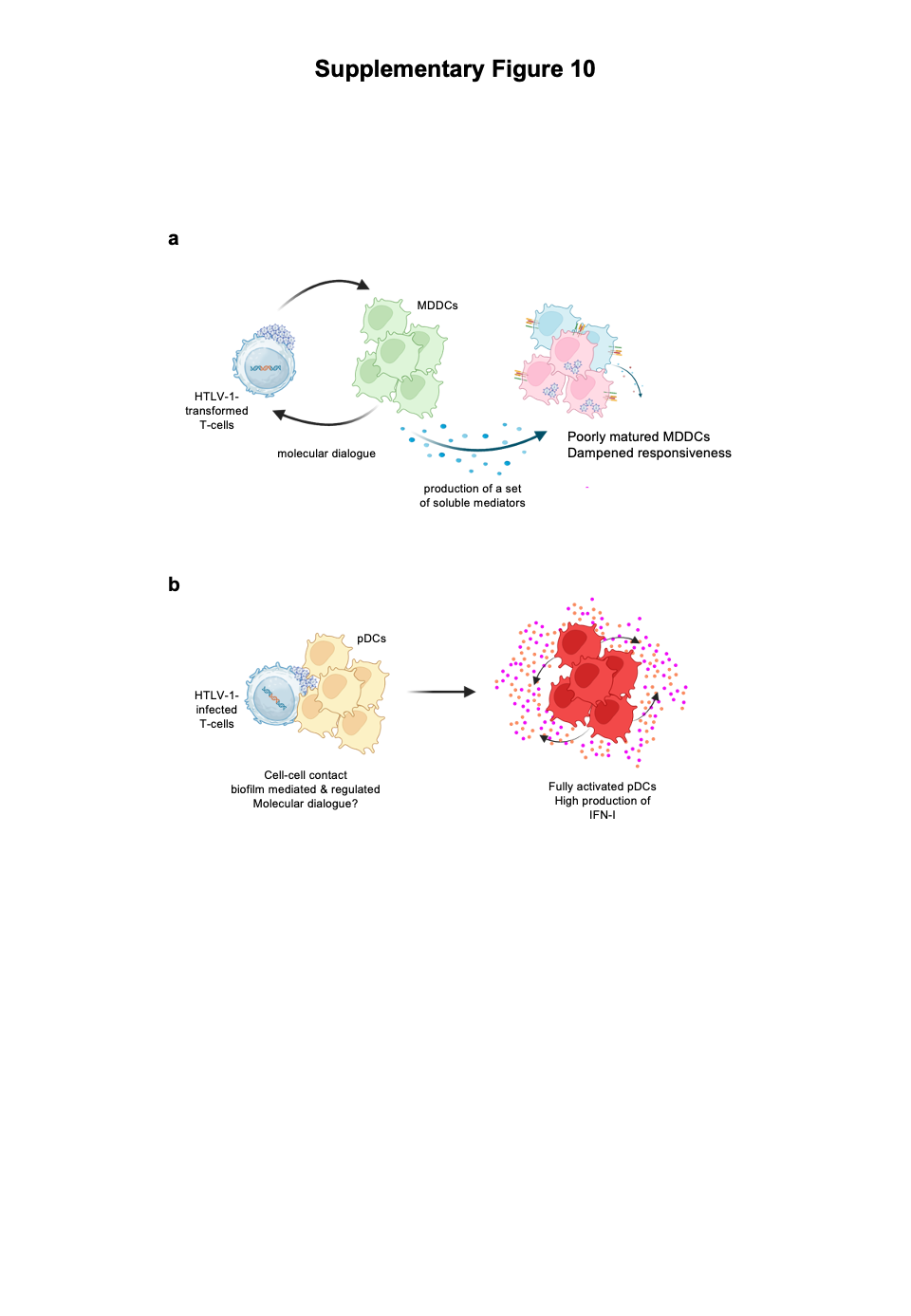

Supplement: S10 Fig — a. Summary of the results presented in this study: a molecular dialogue established between MDDCs and HTLV-1-transformed T-cells upon coculture, partially dependent on viral capture and cell-cell contact, leads to the release of soluble mediators that impair MDDCs maturation and dampen their responsiveness. b. Summary of pDCs response to exposure of HTLV-1-infected cells as reported in [21]: pDCs senses HTLV-1 enveloped virions after contact with HTLV-1-infected T-cells mediated by HTLV-1 biofilm. This results in TLR7-dependent pDCs activation and high production of IFN-I, the amount of which is regulated by biofilm composition. The drawing was created using BioRender.com. (TIFF) [file ppat.1012555.s010.tiff]
