# Supplementary material for: Peculiar transcriptional reprogramming with functional impairment of dendritic cells upon exposure to transformed HTLV-1-infected cells
Source: PLoS Pathog. 2024 Sep 16;20(9):e1012555. doi: 10.1371/journal.ppat.1012555 (PMC11426526; doi:10.1371/journal.ppat.1012555)
Supplement: Attachment — Submitted filename: Rebuttal-Carcone et al.docx [file ppat.1012555.s019.docx]

Dear Editor,

Please find enclosed the revised version of our manuscript entitled “**Peculiar transcriptional reprogramming with functional impairment of dendritic cells upon exposure to transformed HTLV-1-infected cells**” that we submitted as an article for publication in PLoS Pathogens. Responses to reviewers' comments are listed below.

**Minor comments:**

Reviewer #2:

1. Jurkat is equal to C91-PL in Fig. 1b, this is a bit weird as a starting point since the manuscript focuses on HTLV-1-infected T-cells (although later on strong differences are shown in transcriptomics).

We thank the reviewer for this accurate observation. In fact, the comparison between MDDCs exposed to Jurkat and to C91-PL cells is described both in Fig. 1b, and in Fig. 1c (green vs dark blue bars), with independent repeats. Because the difference between the percentage means between both conditions is small (approx. 15 vs 20%), detecting a statistically significant difference requires an increased number of repeats. This may explain why the difference is not significant in Fig. 1b (n=3), while it becomes significant in Fig. 1e (n=15). Our biological interpretation is that there is indeed a difference between Jurkat and C91-PL, but that the magnitude of this difference is very small in comparison to other treatments (MeV or LPS). As indicated by the reviewer, this interpretation is consistent with transcriptomic data provided in Fig. 2.

The text has been clarified accordingly (L120-121, and L174-179).

Likewise, the statistical analysis of Fig. 1 as detailed in Suppl. Table 7 takes Jurkat as a basis for the multiple comparison correction. While the statistical significance will probably not change, it is important that the statistical tests reflect the research hypothesis, i.e. testing the effect of HTLV-1-infected cells rather than uninfected Jurkat cells. The same goes for other multiple-comparison tests in the manuscript.

We agree that depending on how the hypothesis is formulated, using infected cells as a basis for multiple comparisons may be more accurate. This analysis has been added for all relevant panels in Fig. 1 (see **Supplementary Table 7**). The legend of Fig. 1 has also been clarified (L155-170). As foreseen by the reviewer, this does not change the statistical significance.

Statistical analysis of **Supplementary Fig. 2** has also been complemented by a nested analysis, grouping cell lines according to their infection status (see **Supplementary Table 8**). The legend of **Supplementary Fig. 2** has also been clarified accordingly.

For multiple comparisons in restimulation experiments (**Supplementary Fig. 5**), Jurkat-exposed MDDCs stimulated with LPS are used as the reference, and each infected cell line is compared to this reference (see **Supplementary Table 8**).

The text of the related Material and Methods section has been updated accordingly (L849-851, and 861-869).

2. About the role of ISG and type I IFN: the top 100 most upregulated genes in Sup Table 1 are almost exclusively ISG, several of which are present in the HAM disease signature (Tattermusch et al.). As outlined above, I feel the difference between C91-PL and Jurkat is an important finding that merits more discussion. Several of the genes revealed in the transcriptomic analysis across the manuscript FAS/STAT1 and ISGs are found in HAM, especially IL17 signaling has been shown by several groups (Leal et al., Assone et al…..) and confirmed by a recent systematic review (Shafiei et al. Cytokine 2024). Please compare your proposed signature to published HTLV-1 transcriptomic datasets (publicly available) of other groups in either HTLV-1 infected T-cells (Moens et al., Kashima group from Brazil), and particularly in HAM (Tattermusch et al.) and ATL patients (Watanabe group Kataoka et al., Dierckx et al.).

We agree that discussing our findings in comparison to other publicly available transcriptomic datasets is of interest.

As suggested by the reviewer, we first focused on ISGs present in the DEG list in **Supplementary Table 1** (Jurkat-exposed MDDCs vs. C91-PL-exposed MDDCs). Among the 372 known ISG, only 121 were retrieved in the set of significantly upregulated genes (please note that the number of known ISG was wrong in the previous version of the manuscript: it has now been corrected in L271). When analyzing the top 100 most upregulated genes (filtered on the fold change value), only 18 ISG were retrieved. We thus do not come up with the same observation as the reviewer ("the top 100 most upregulated genes in Sup Table 1 are almost exclusively ISG").

Nonetheless, we compared these 18 ISG with the signature described by Tattermusch et al., 2012 (PMID: 22291590). In this paper, the authors analyzed the DEG in the blood from HTLV-1-infected individuals (both asymptomatic carriers and HAM patients), compared to the blood from seronegative individuals, as well as the DEG specific to HAM patients. When comparing the ISG retrieved in our analysis of HTLV-1-exposed MDDCs with the ISG retrieved by Tattermusch et al., we obtained only 7 ISG in common with the signature of HTLV-1 infection, among which only 3 ISG in common with the signature of HAM.

When extending the analysis to the 474 DEG from **Supplementary Table 1**, including 121 ISG as stated above, we retrieved 25 ISG in common with the signature of HTLV-1 infection, among which 9 ISG in common with the signature of HAM. A total number of 36 DEG (ISG and others) were found common between our dataset and the signature of HTLV-1 infection.

We performed a similar comparison with the datasets from Pinto et al., 2014 (PMID: 24041428) and Malta et al., 2013 (PMID: 23301858), from the Kashima group. In these papers, the transcriptional profiles of CD4+ and CD8+ T cells, respectively, from asymptomatic carriers and HAM patients were analyzed. When comparing their lists of DEG with the one we obtained, only very few genes in common were retrieved (0-3 depending on the dataset).

We also considered other publicly available datasets, for instance from Moens et al., 2012 (PMID: 22917064), from Khouri et al., 2018 (PMID: 29721391) or from Leal et al., 2018 (PMID: 29872426). In these papers, genes differentially expressed upon type I IFN treatment of HTLV-1-infected cell lines, or of PBMCs from ATL or HAM patients, respectively, were identified. However, comparison with our dataset did not lead to any clear conclusion, probably because in our approach, we tested the impact of exposure to infected vs non-infected cells, rather than the response to IFN treatment in the context of infection.

As suggested by the reviewer, and in line with the data from Assone et al., 2022 (PMID: 36482436) and the review by Shafiei et al., 2024 (PMID: 39089216), we then focused on the IL-17 signaling pathway. In our manuscript, we had identified this pathway as a KEGG pathway over-represented among the down-regulated genes upon restimulation (MDDC/C91-PL restim w/ LPS vs MDDC/Jurkat restim w/ LPS, **Fig. 4b-c**). This can be interpreted as the fact that pre-exposure to HTLV-1 dampens the ability of naive MDDCs to induce the IL-17 pathway upon stimulation by a TLR agonist.

To gain insight into the modulation of the IL-17 pathway in MDDCs upon contact with HTLV-1-infected cells (corresponding to the experimental set-up of **Fig. 2**), we retrieved the list of genes annotated in the IL-17 KEGG pathway among the 474 DEG listed in **Supplementary Table 1**. Three such genes were identified, and all 3 were upregulated by the contact with HTLV-1-infected cells. This is indeed consistent with the IL-17 signature associated with progression to HAM in HTLV-1-infected individuals.

While these comparisons help us integrate our findings in the context of the HTLV-1 literature, we ought to keep in mind that our experimental settings are very distinct from the ones analyzed in other papers. Among the differences, the following are noteworthy:

- We present the transcriptomic signature of MDDC exposed to infected or non-infected T cell lines, while most other studies investigate the transcriptomic profile of total blood, PBMCs, or purified CD4+ or CD8+ T cells, from infected individuals, or from T cell lines. Thus, the cell type is different among studies, and this could lead to distinct transcriptomic profiles in terms of individual DEGs (although consistent at the systemic level). For instance, myeloid dendritic cells only represent approx. 0.2% of whole blood cells in Tattermusch et al.
- In addition, we assess the acute transcriptomic response of MDDCs upon exposure to infected cell lines, while most other studies analyze the profile of cells from chronically infected individuals (be they asymptomatic carriers, HAM or ATL patients) or T cell lines. Thus, the kinetics of exposure to HTLV-1 is very different between reports.

Altogether, the biological interpretation of such comparisons should be taken with great caution.

These analyses have been added in **Supplementary Table 1**, and discussed in the manuscript (L656-672).

3. A minor follow-up question: can the proposed signature be tolerogenic if ISG and (at least a partial) IFN signature is detected?

This is a good point. We observe that the induction of ISG in MDDCs upon exposure to HTLV-1 is very limited compared to LPS stimulation. This holds true both in terms of number of ISG induced (only 121 genes among 372), and in terms of amplitude of expression (their upregulation is lower than that induced by LPS stimulation, see **Supplementary** **Fig. 4b**). We thus suggest that such a poor induction of ISG is not sufficient to confer functional responsiveness to MDDCs. In combination with the induction of a specific transcriptional signature in HTLV-1-exposed MDDCs (see **Fig. 2d**), this poor induction of ISG could allow a tolerogenic behavior in MDDCs.

This was discussed in the manuscript (L617-625 and 636).

Reviewer #3:

I suggest a graphical representation on the effect of HTLV-1 on pDC and mDC.

We thank the reviewer for this suggestion. Such a representation was added as **Supplementary Fig. 10**, and called at L568-569).
